# New Solid-State Acoustic Motion Sensors: Sensing Potential Estimation for Different Piezo Plate Materials

**DOI:** 10.3390/s24134271

**Published:** 2024-07-01

**Authors:** Michail Shevelko, Andrey Baranov, Ekaterina Popkova, Yasemin Staroverova, Aleksandr Peregudov, Alexander Kukaev, Sergey Shevchenko

**Affiliations:** 1 Department of Electroacoustics and Ultrasonic Technology, Saint Petersburg Electrotechnical University, Saint Petersburg 197002, Russia; mmshevelko@etu.ru (M.S.); adbaranov@etu.ru (A.B.); yadurukan@etu.ru (Y.S.); anperegudov@etu.ru (A.P.); 2 Department of Laser Measurement and Navigation Systems, Saint Petersburg Electrotechnical University, Saint Petersburg 197002, Russia; askukaev@etu.ru (A.K.); syshevchenko@etu.ru (S.S.)

**Keywords:** acoustic motion sensor, solid-state sensitive element, bulk acoustic wave, sensing potential, parameter optimization, sensitive measurement

## Abstract

The present paper discusses the scientific and technical problem of optimizing the design and characteristics of a new type of solid-state sensors for motion parameters on bulk acoustic waves in order to increase the signal-to-noise ratio and the detectability of an informative signal against the background of its own noise and interference. Criteria for choosing materials for structural elements, including piezoelectric transducers of the sensitive element, were identified; a corresponding numerical simulation was performed using the developed program; and experimental studies according to the suggested method were carried out to validate the obtained analytical and calculated positions. The experimental results revealed the correctness of the chosen criteria for the optimization of design parameters and characteristics, demonstrated the high correlation between the results of modeling and field studies, and, thus, confirmed the prospects of using this new type of solid-state acoustic sensors of motion parameters in the navigation and control systems of highly dynamic objects.

## 1. Introduction

Ultrasonic sensors are widely used in various fields of modern science and technology, such as medicine, non-destructive testing, hydroacoustics, acoustoelectronics, etc. [1,2,3,4,5,6,7,8]. In particular, one of the actual directions of the development of ultrasonic sensors is in navigation and motion control, where acoustic sensors can compete with MEMS [9,10].

Attempts to mathematically describe the process of the propagation of acoustic waves in the presence of the rotation of the acoustic duct medium were made by a number of authors [11,12,13]. However, a few decades later, the group of authors of the present paper managed to find the acoustic wave parameter associated with the angular velocity of rotation, to formulate concepts for designing a new type of acoustic-sensitive elements of sensors, and to obtain an experimental confirmation of the detectability of an informative signal for their developed model of the solid-state sensitive element [14,15].

In recent years, the development of a new generation of solid-state sensitive elements of motion parameter sensors has been carried out at the Department of Electroacoustics and Ultrasonic Technology of the St. Petersburg Electrotechnical University ETU “LETI”. An informative signal of this type of sensor is a change in the parameters of acoustic waves propagating in a solid medium in the presence of rotation. The authors have offered a number of concepts for the constructive implementation of such sensitive elements [14,15,16]. Their advantage over MEMS is the ability to work in highly dynamic objects under high load due to the absence of masses on torsion bars in the design.

In this case, a separate important task in the development of solid-state acoustic sensors on bulk acoustic waves is to increase their sensing potential, which places high demands on the materials used and also forms criteria for selecting the appropriate cuts.

**Figure 1:**
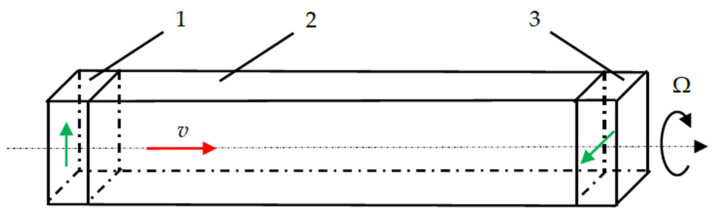
Sensitive element of solid-state acoustic gyro sensor with rotated transducers: 1—emitting piezoelectric plate; 2—acoustic duct; 3—receiving piezoelectric plate.

The paper [14] considers the principle of constructing a sensitive element of a gyroscope based on a change in the polarization of the emitted transverse acoustic wave under the Coriolis force as it propagates in a rotating acoustic duct, shown in Figure 1, where *v* is a phase velocity of the bulk acoustic wave and Ω is a rotation velocity. In this case, the amplitude of the resulting orthogonal component due to rotation is proportional to the angular velocity of rotation.

To detect the informative signal, the emitting and receiving transducers (two piezoelectric plates rotated relative to each other by 90°) are glued at the opposite ends of the solid-state acoustic duct.

**Figure 2:**
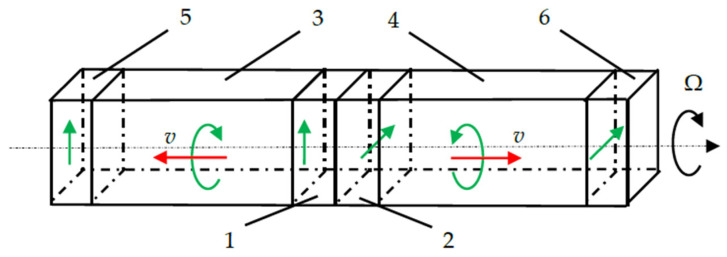
Sensitive element of solid-state acoustic gyro sensor based on circular polarized waves: 1, 2—emitting piezoelectric plates; 3, 4—acoustic ducts; 5, 6—receiving piezoelectric plates.

The work [16] considers the principal design of the sensitive element of a gyroscope based on the propagation in opposite directions of two circularly polarized waves in a rotating solid medium, as shown in Figure 2.

To emit circularly polarized waves, a complex transducer is used, consisting of two piezoelectric plates with orthogonal polarization directions. The informative signal is the phase difference between two waves received at opposite ends of identical sound ducts, proportional to the angular velocity of rotation.

To implement the concepts described above for constructing sensitive elements of motion parameter sensors, as well as to detect an informative signal proportional to the angular velocity of rotation against a background of self-noise and interference, the piezoelectric transducers must ensure the effective emission and receiving of a purely transverse wave at frequencies above 10 MHz to achieve sensor miniaturization.

Plate piezoelectric transducers are widely used to excite both longitudinal and transverse waves. In this case, it is necessary to provide an effective emission of acoustic waves based on the criteria for their further application: for non-destructive testing, sounding depth and resolution; for medicine, operation in an environment with different levels of sound absorption; for flow metering, accuracy in determining flow velocity; and for sensorics, the required sensitivity. When solving such tasks in these areas, the standard industrially produced longitudinal wave transducers with operating frequencies up to 10 MHz are commonly used, and the main criterion is directly the efficiency of electromechanical transformation that depends on the components of the piezo constant tensor of the material.

However, to provide the operation of solid-state acoustic motion sensors under consideration, it is of great practical interest to find specific cuts of piezodielectric materials to make the piezo plate that provides not only the effective conversion of electrical energy to mechanical vibration one but also the effective generation of bulk acoustic waves of purely transverse polarization at high frequencies.

Thus, the objective of this paper is to formulate, on the basis of theoretical analysis, criteria for the selection of optimal piezoelectric materials and their cuts for the sensitive element of solid-state acoustic gyro sensors that provide both high efficiency in the generation of bulk acoustic waves of the transverse polarization as well as a further experimental confirmation of the obtained scientific–technical statements.

## 2. Numerical Analysis

### 2.1. Theoretical Basis

To reveal the cuts of piezoelectric plates that can be used to effectively excite and receive bulk acoustic waves of various polarizations, the new method was developed, and this consists of analyzing the angular dependences of the characteristics of bulk acoustic waves propagating into the piezodielectric material. The theoretical basis for this numerical analysis is the theory of crystal acoustics.

One of the informative parameters to reveal the direction of pure polarized wave propagation comprises the angular dependences of phase velocities. Phase velocities for the selected direction $\vec{l}\left(l_{1},l_{2},l_{3}\right)$ are defined as the eigenvalues of the Green–Christoffel tensor [17]:

(1) $$\left|Q_{im}^{*}-\rho \omega^{2}\delta_{im}\right|=0,$$

Here, $Q_{im}^{*}=C_{iklm}l_{k}l_{l}$ is a Green–Christoffel tensor, *C_iklm_* is an elastic modulus [17,18,19,20], ρ is the material density, ω is the radial frequency, and δ*_im_* is a Kronecker symbol.

To estimate the efficiency of electroacoustic transformation in the direction perpendicular to the cut of the piezodielectric material, the electromechanical transformation coefficient *K_t_* is considered. In a given direction, the electromechanical transformation coefficient depends on wave propagation velocities while and without taking into account the piezoelectric effect [21]:

(2) $$K_{t}=\sqrt{\frac{\left|v_{p}^{2}-v^{2}\right|}{v^{2}}},$$

Here, *v_p_* is the wave propagation velocity while taking into account the piezoelectric effect; *v* is the wave propagation velocity without taking into account the piezoelectric effect.

The velocity *v* in a piezodielectric crystal is found by taking the tensor of the piezoelectric constants to be equal to zero, that is, a dielectric material is considered with identical elastic properties.

Another fundamentally important criterion for choosing a material and cut for a piezoelectric plate is the angle ψ between the propagation direction of the quasi-longitudinal wave and its polarization vector. When the angle ψ is zero, the wave is purely longitudinal, which means that the other two waves propagating in a given direction are purely transverse [17]. In turn, the radiation of a purely transverse wave is a necessary condition for the performance of the solid-state acoustic motion sensor under consideration. This situation is not typical for other types of sensors, the operation of which usually allows the existence of both transverse and longitudinal bulk acoustic waves, and the radiation of two transverse waves is acceptable.

The calculation program is developed using MATLAB 2023a to plot the graphic dependances of wave phase velocities *v*, electromechanical transformation coefficient *K_t_*, and angle ψ for quasi-longitudinal waves on the direction of wave propagation in piezodielectric materials.

To estimate the phase velocity in a certain direction, the eigenvalues and then the eigenvectors of the Christoffel tensor are to be found that correspond to the wave polarization vectors. Thus, the angular dependence of the eigenvalues of the Christoffel tensor is formed since its components depend on the direction under consideration. This task is a non-trivial one due to the presence of singular points—some directions in which a pair of eigenvalues are equal to each other so that sets of eigenvalues with equal values are mixed, which interferes with the analysis of the resulting curves.

**Figure 3:**
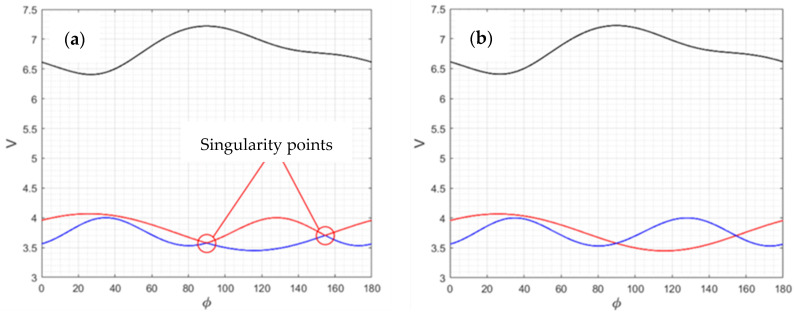
Singularity points: (**a**)—incorrect phase velocity curves plotting; (**b**)—correct phase velocity curves plotting.

When calculating the eigenvalues and eigenvectors of the Green–Christoffel tensor, the built-in function eig is used, and this returns the eigenvalues and eigenvectors in some random order. At singularity points, where the phase velocity values are equal to each other and two velocity curves intersect, the eig function changes the order of the returned values so that the resulting phase velocity curves are mixed with each other, as shown in Figure 3. This makes it impossible to find a valid correspondence between the velocity and polarization of the excited waves and, therefore, does not allow the correct selection of the material and cut of piezoelectric plates to ensure the operation of the sensor.

To overcome this issue, a specific algorithm is implemented: each iteration of eigenvalue and eigenvector calculation is carried out while taking into account the previously obtained values. In this case, the resulting values are rearranged so that each subsequent resulting value is as consistent as possible with the previous one. Also, since the calculation of eigenvectors can be performed that are accurate to the sign, the sign applied to the resulting eigenvector is chosen to match, as closely as possible, the preceding vector in the sequence of eigen tasks.

Thus, the correct sets of phase velocity curves are obtained, as are the values of the polarization vectors, by performing a rearrangement, taking into account the maximum correspondence of the calculated values from each sequence step to the next one, as well as taking into account the anisotropic properties of the material.

The following sections present the results of numerical modeling for a number of materials that are promising for use in solid-state acoustic gyroscopes, as well as an analysis of the data obtained in order to find optimal cuts and directions to emit and receive bulk acoustic waves of pure polarization.

### 2.2. Piezo Quartz

**Figure 4:**
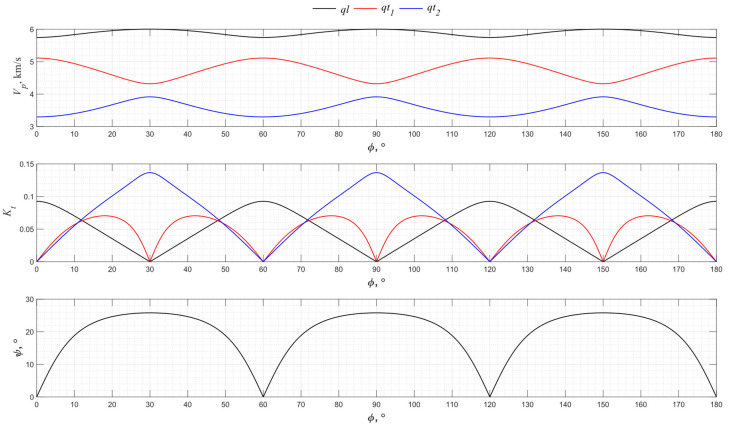
The propagation velocity *v_p_*, electromechanical transformation coefficient *K_t,_* and angle ψ angular dependencies for the *XY* cut of piezo quartz.

The numerical modeling results for the angular dependence of the propagation velocity values of bulk acoustic waves of various polarizations for the *XY* cut of piezo quartz are shown in Figure 4, where *ql* is the quasi-longitudinal wave and *qt*_1_ and *qt*_2_ are the quasi-transverse waves.

From the presented graphs, one can conclude that the directions of 0°, 60°, and 120° for the *XY* cut provide the excitation of purely longitudinal waves associated with the piezoelectric effect. The *X*-cut quartz plate represents a classic method for exciting longitudinal waves. Directions 30°, 90°, and 150° have the maximum piezoelectric effect associated with only one of the transverse waves. Thus, the use of a *Y* cut allows transverse acoustic waves to be emitted into an isotropic medium.

**Figure 5:**
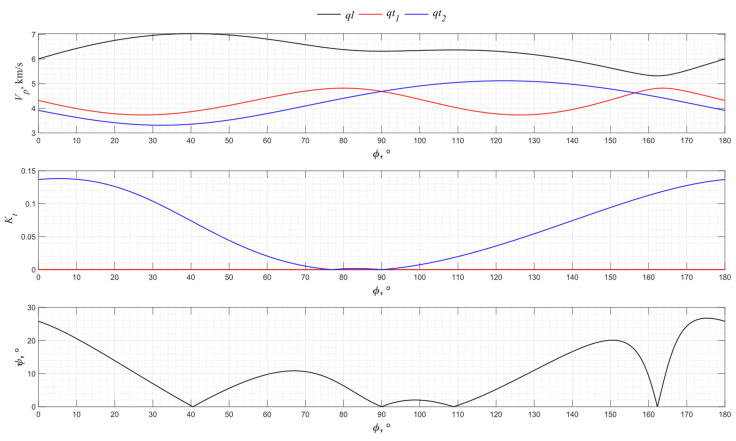
The propagation velocity *v_p_*, electromechanical transformation coefficient *K_t_*, and angle ψ angular dependencies for the *YZ* cut of piezo quartz.

Similar graphs for the angular dependence of the propagation velocity of bulk acoustic waves of various polarizations for the *YZ* cut of piezo quartz are shown in Figure 5.

From the presented graphs, one can see that only one quasi-transverse wave has a piezoelectric effect in a given cut. The direction along the *Z* axis is non-piezoelectric. The maximum excitation efficiency corresponds to the *Y* + 5° direction.

**Figure 6:**
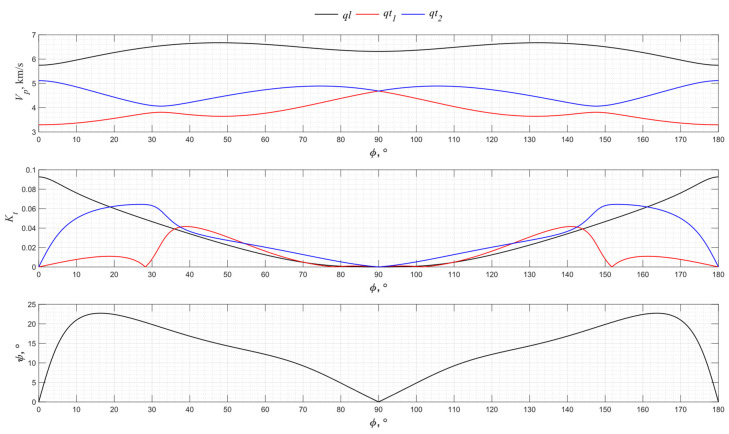
The propagation velocity *v_p_*, electromechanical transformation coefficient *K_t_*, and angle ψ angular dependencies for the *XZ* cut of piezo quartz.

The graphs for the angular dependence of the propagation velocity of bulk acoustic waves of various polarizations for the *XZ* cut of piezo quartz are shown in Figure 6.

From Figure 6, it can be seen that the *X* axis is the most effective direction for longitudinal wave excitation. The *Z* axis, in which direction a purely longitudinal and, accordingly, two purely transverse waves can propagate, has no piezoelectric effect. Directions 27° and 153° in this cut have the highest efficiency of excitation of one of the quasi-transverse waves, accompanied by the piezoelectric effect of the quasi-longitudinal wave. In directions 29° and 151°, there is no piezoelectric effect for one of the quasi-transverse waves.

### 2.3. La_3_Ga_5_SiO_14_

**Figure 7:**
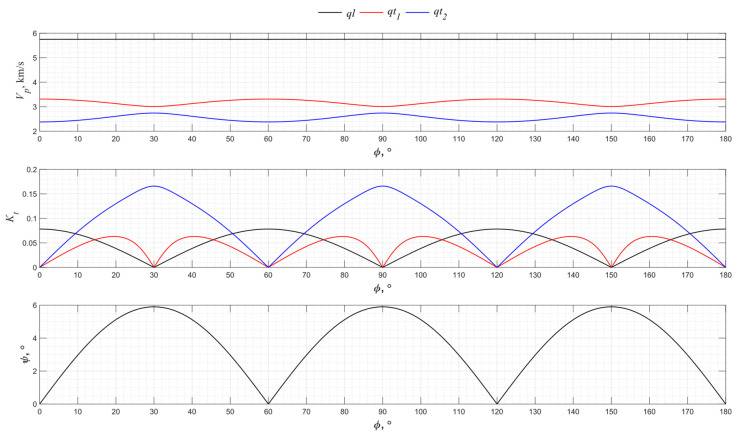
The propagation velocity *v_p_*, electromechanical transformation coefficient *K_t_*, and angle ψ angular dependencies for the *XY* cut of La_3_Ga_5_SiO_14_.

The numerical modeling results for the angular dependence of the propagation velocity of bulk acoustic waves of various polarizations for the *XY* cut of langasite La_3_Ga_5_SiO_14_ are shown in Figure 7.

Langasite is of the same crystallographic symmetry class as a piezo quartz, so the directions 0°, 60°, and 120° of the *XY* cut allow the generation of pure transverse waves, as do the 30°, 90°, and 150° directions. The efficiency of the excitation of pure waves in langasite is higher than in piezo quartz due to the highest electromechanical transformation coefficient of La_3_Ga_5_SiO_14_.

**Figure 8:**
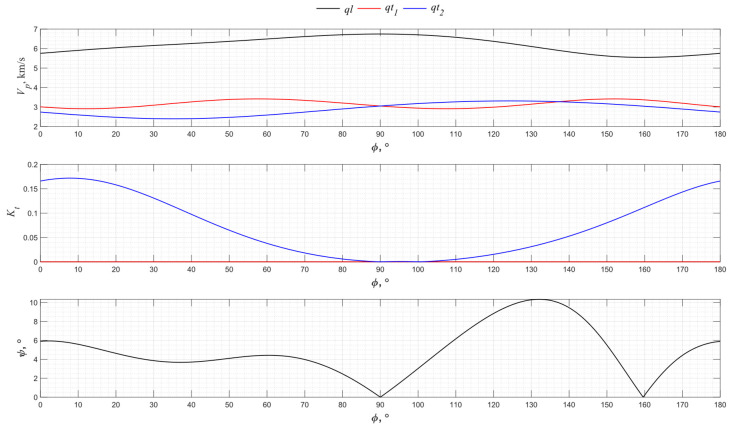
The propagation velocity *v_p_*, electromechanical transformation coefficient *K_t_*, and angle ψ angular dependencies for the *YZ* cut of La_3_Ga_5_SiO_14_.

Similar graphs for the angular dependence of the propagation velocity of bulk acoustic waves of various polarizations for the *YZ* cut of langasite are shown in Figure 8.

The most efficient transverse wave excitation for langasite in the *YZ* plane is achieved by using a cut orthogonal to the 7.8° direction. The *Z* cut of langasite, as well as the similar direction in piezo quartz, has no piezoelectric properties.

**Figure 9:**
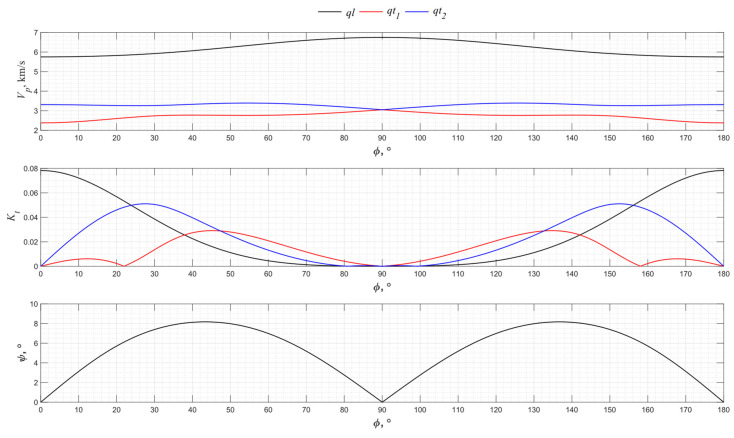
The propagation velocity *v_p_*, electromechanical transformation coefficient *K_t_*, and angle ψ angular dependencies for the *XZ* cut of La_3_Ga_5_SiO_14_.

The graphs for the angular dependence of the propagation velocity of bulk acoustic waves of various polarizations for the *XZ* cut of langasite are shown in Figure 9.

From Figure 9, it can be seen that in the *XZ* plane, in directions 21° and 159°, it is possible to excite one quasi-transverse and one quasi-longitudinal wave since one of the quasi-transverse waves is not associated with the piezoelectric effect. In directions 27.5° and 152.5°, the maximum excitation of one of the quasi-transverse waves is reached. Also, the plots built for the *XZ* plane confirm the previously made conclusions about the *X* and *Z* cuts: the *X* cut is the direction with the greatest efficiency of excitation of longitudinal waves and the *Z* cut has no piezoelectric effect.

### 2.4. LiNbO_3_

**Figure 10:**
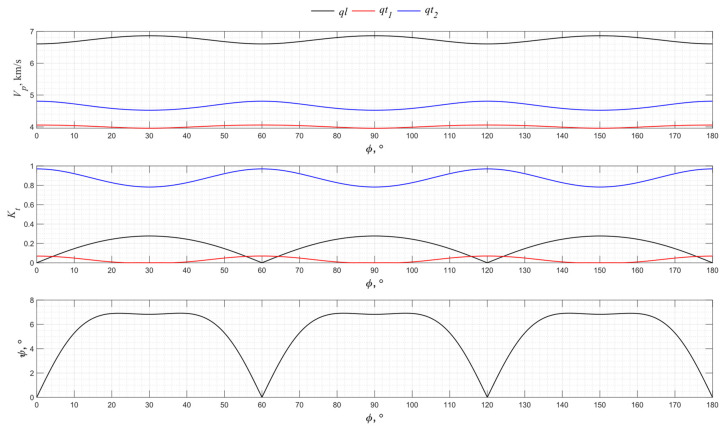
The propagation velocity *v_p_*, electromechanical transformation coefficient *K_t_*, and angle ψ angular dependencies for the *XY* cut of LiNbO_3_.

The numerical modeling results for the angular dependence of the propagation velocity of bulk acoustic waves of various polarizations for the *XY* cut of lithium niobate LiNbO_3_ are shown in Figure 10.

The *X* cut of lithium niobate is widely used to excite the transverse waves. However, the calculation result shows that in directions 0°, 60°, and 120°, due to the piezoelectric effect, two purely transverse waves are excited. At the same time, the coefficient of electromechanical transformation of the first transverse wave is ten times higher than of the second one, which indicates the elliptical nature of the wave excited into an isotropic medium. In directions 30°, 90°, and 150°, due to the piezoelectric effect, one quasi-transverse wave and one quasi-longitudinal wave are excited.

**Figure 11:**
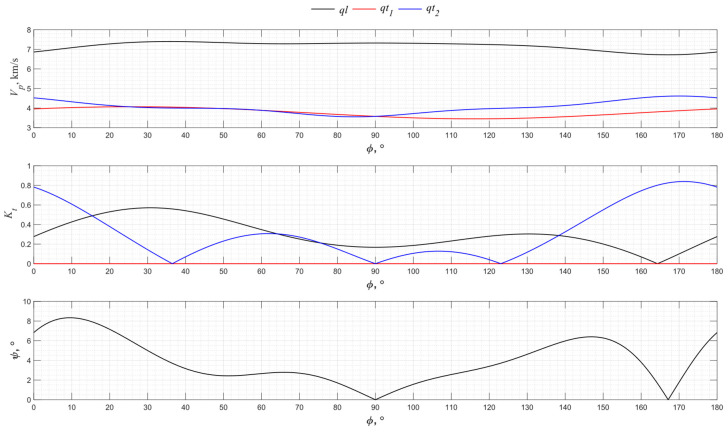
The propagation velocity *v_p_*, electromechanical transformation coefficient *K_t_*, and angle ψ angular dependencies for the *YZ* cut of LiNbO_3_.

Similar graphs for the angular dependence of the propagation velocity of bulk acoustic waves of various polarizations for the *YZ* cut of lithium niobate are shown in Figure 11.

In the *YZ* plane of lithium niobate, the piezoelectric effect does not affect one of the quasi-transverse waves. The highest efficiency of quasi-transverse wave excitation is achieved in the direction 171°. In the direction 164°, due to the piezoelectric effect, a quasi-longitudinal wave is not excited. In the direction 167°, the generated waves acquire purely transverse and purely longitudinal polarization. The excitation of only quasi-longitudinal waves is possible in directions of 36° and 123° and along the Z axis.

**Figure 12:**
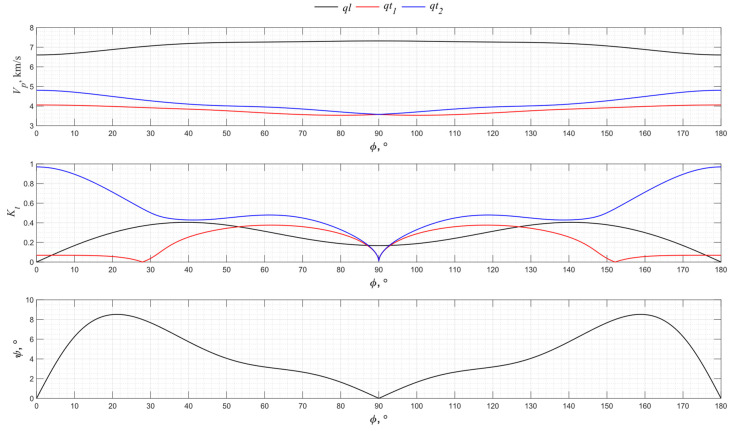
The propagation velocity *v_p_*, electromechanical transformation coefficient *K_t_*, and angle ψ angular dependencies for the *XZ* cut of LiNbO_3_.

The graphs for the angular dependence of the propagation velocity of bulk acoustic waves of various polarizations for the *XZ* cut of lithium niobate are shown in Figure 12.

From Figure 11, it can be seen that in the *XZ* plane of lithium niobate, the highest value of the electromechanical transformation coefficient is achieved in the directions of 61° and 119°. In directions 28° and 152°, due to the piezoelectric effect, one quasi-transverse wave and one quasi-longitudinal wave are generated. The *Z* cut of lithium niobate allows only pure longitudinal waves to be excited.

### 2.5. PZT-5H

In order to study the possibility of using piezoceramics as materials in acoustic solid-state gyro sensors, wave propagation in PZT-5H ceramics was also analyzed. Piezoceramics are polycrystalline materials, so they should be considered for analysis as media with transverse isotropy. The direction of piezoceramic polarization is taken to be the *Z* axis.

**Figure 13:**
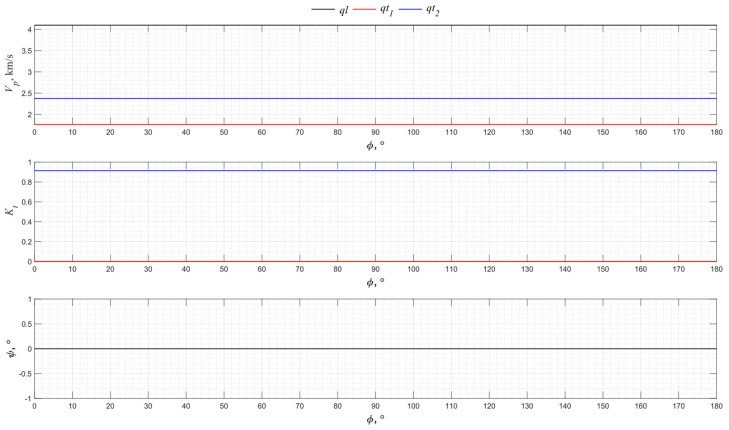
The propagation velocity *v_p_*, electromechanical transformation coefficient *K_t_*, and angle ψ angular dependencies for the *XY* cut of PZT-5H.

Figure 13 shows the corresponding graphs for the *XY* plane for PZT-5H piezoceramics.

From the graphs in Figure 13, it is seen that regardless of the selected cut, which normally lies in the *XY* plane, the piezoelectric plate will excite a pure transverse wave with high efficiency.

Due to the transverse isotropy of piezoceramics, the results obtained in the *XZ* and *YZ* planes are identical.

**Figure 14:**
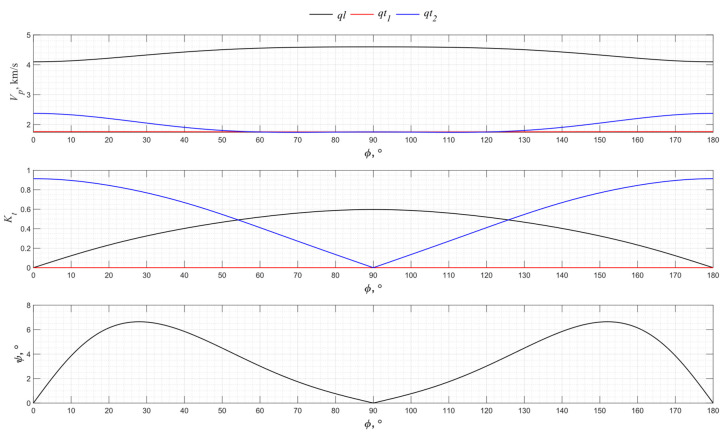
The propagation velocity *v_p_*, electromechanical transformation coefficient *K_t_*, and angle ψ angular dependencies for the XZ-cut and *YZ* cut of PZT-5H.

Figure 14 shows the corresponding graphs for the *XZ* and *YZ* planes for PZT-5H piezoceramics.

From the graphs in Figure 14, it can be concluded that the *Z* direction, which is the direction of piezoceramic polarization, has the largest relative velocity increment, which makes it possible to use this cut for the effective excitation of purely longitudinal waves.

### 2.6. Discussion

The analysis of the obtained results from numerical simulation as graphical dependencies of the bulk acoustic wave velocities, as well as the coefficient of electromechanical transformation, in various media allows to conclude that it is possible to identify a number of materials, cuts, and directions wherein the propagation of pure polarized waves is observed, which makes them optimal for use as materials for the acoustic sensor for the further detecting and processing of an informative signal.

The next stage in a complex study is to verify the obtained results of a numerical modeling of materials using experimental research to confirm their applicability in practice for the designing of sensitive elements of acoustic solid-state gyro sensors.

## 3. Experimental Setup

**Figure 15:**
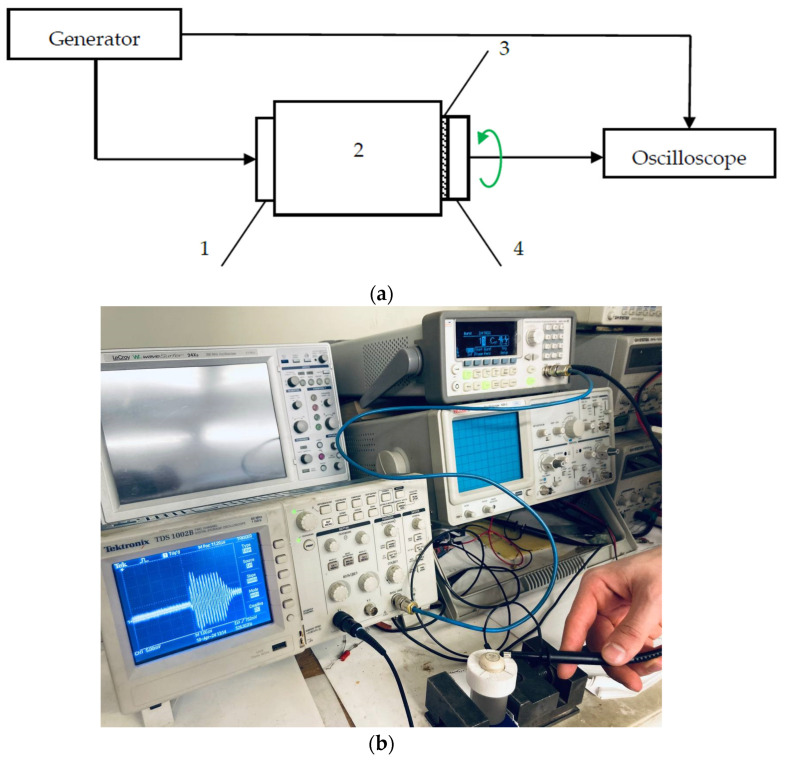
The experimental setup for studying the polarization of acoustic waves: (**a**) block diagram: 1—metallized radiating piezodielectric plate; 2—isotropic acoustic duct; 3—contact layer; 4—metallized receiving piezodielectric plate; (**b**) photograph of the experimental setup.

To study the polarization of waves emitted by piezodielectric plates of materials and cuts that provide purely transverse and purely longitudinal waves according to the numerical calculations obtained, an experiment was carried out, according to methodology described below, using a test setup, the structural diagram of which is shown in Figure 15.

## 4. Experimental Results

During test series, four pairs of emitting and receiving plates made of *Y*-cut piezo quartz, *Y*-cut langasite, shear cut ceramic, and *X*-cut lithium niobate were used. The acoustic duct was made of fused quartz.

Firstly, to operate in the passband of the acoustic system 1-2-3-4, which ensures the maximum transmission coefficient, the frequency response for each pair was studied with a coaxial orientation of the sensitivity axes of the emitting and receiving piezoelectric plates. Then, the main part of the research with a rotating receiving plate was carried out to determine the polarization of the radiated acoustic wave and to verify the numerical simulation results.

### 4.1. Piezo Quartz

**Figure 16:**
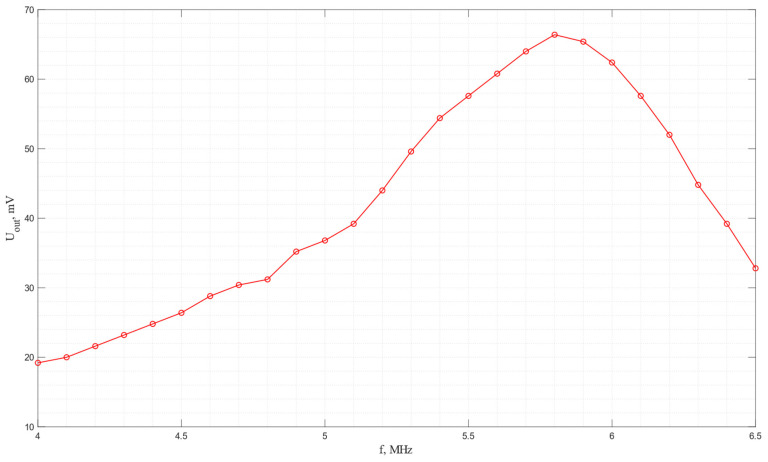
The frequency dependence of the output voltage for the acoustic system with receiving and emitting plates made of *Y*-cut piezo quartz.

The amplitude–frequency characteristic of the acoustic system in which a pair of piezo plates made of *Y*-cut piezo quartz are used is shown in Figure 16. As seen from the graph, the maximum efficiency of electroacoustic transformation is observed at a frequency of 5.8 MHz.

**Figure 17:**
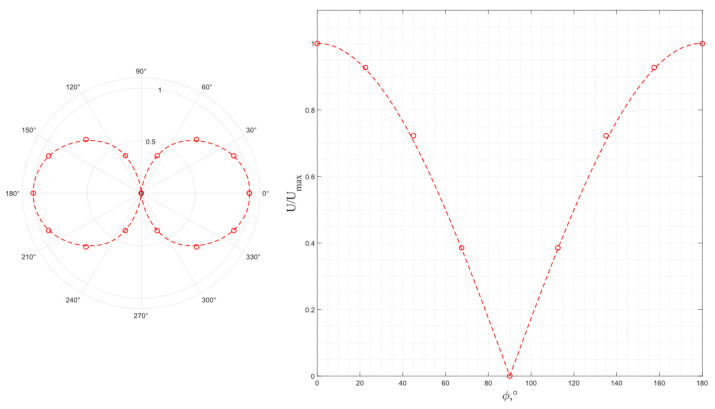
The experimental output voltage’s dependence on the relative orientation of the radiating and receiving piezo plates made of *Y*-cut piezo quartz.

The results of a study of the wave polarization for a pair of piezo plates made of *Y*-cut piezo quartz are shown in Figure 17, where the dotted line is a function, |cos ϕ|. Due to the symmetry to the mutual rotation, the experiment was carried out at rotation angles of 0° ≤ ϕ ≤ 90°. As seen from the graph, the obtained results correspond to the excitation of a single pure transverse wave that matches the numerical simulation results.

The presence of zero with the orthogonal mutual orientation of the sensitivity axes of the plates is also indicated by a phase change of the filling radio pulse on the oscilloscope screen.

### 4.2. La_3_Ga_5_SiO_14_

**Figure 18:**
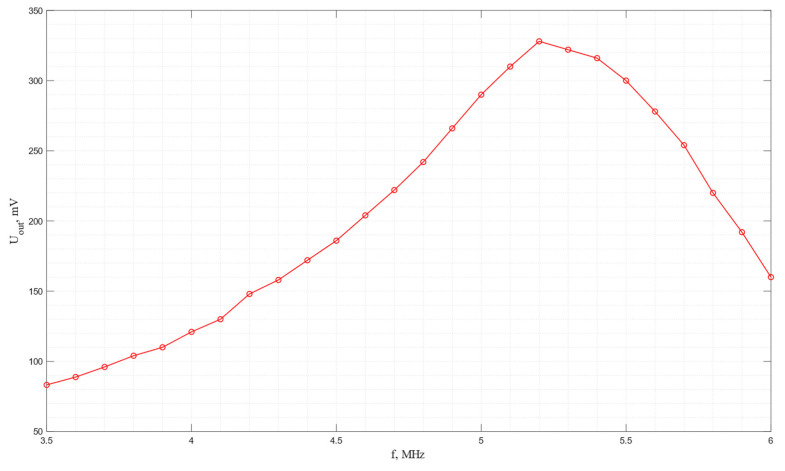
The frequency dependence of the output voltage for the acoustic system with receiving and emitting plates made of *Y*-cut langasite.

The amplitude–frequency characteristic of the acoustic system in which a pair of piezo plates made of *Y*-cut langasite are used is shown in Figure 18. As seen from the graph, the maximum efficiency of electroacoustic transformation is observed at a frequency of 5.2 MHz. In this case, it is important to highlight the higher energy conversion efficiency compared to piezo quartz.

**Figure 19:**
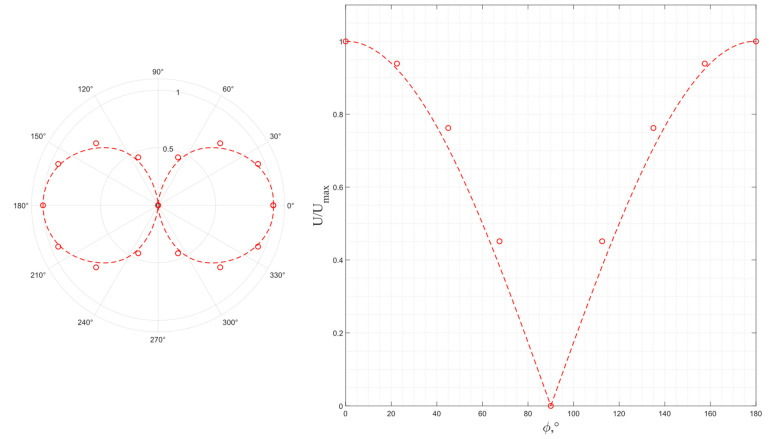
The experimental output voltage’s dependence on the relative orientation of the radiating and receiving piezo plates made of *Y*-cut langasite.

The results of a study of the wave polarization for a pair of piezo plates made of *Y*-cut langasite are shown in Figure 19, where the dotted line is a function, |cos ϕ|. Similar to piezo quartz plates, langasite excites a single pure transverse wave; at the same time, taking into account the graph in Figure 18, the conversion efficiency of langasite is higher, which also corresponds to the calculation results.

### 4.3. LiNbO_3_

**Figure 20:**
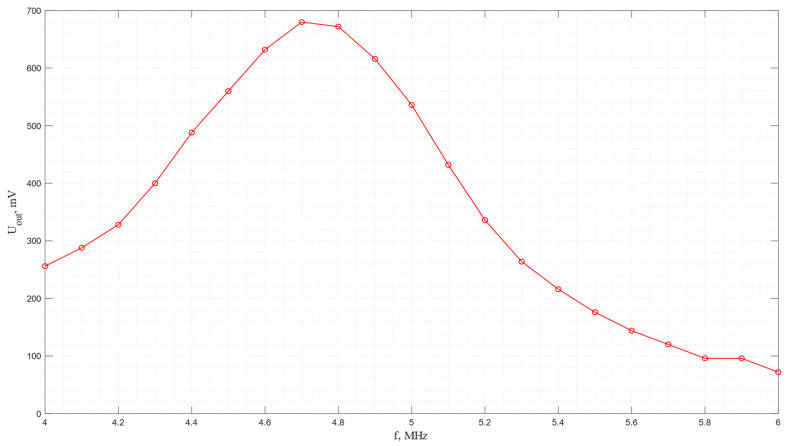
The frequency dependence of the output voltage for the acoustic system with receiving and emitting plates made of *X*-cut lithium niobate.

The amplitude–frequency characteristic of the acoustic system in which a pair of piezo plates made of *X*-cut lithium niobate are used is shown in Figure 20.

**Figure 21:**
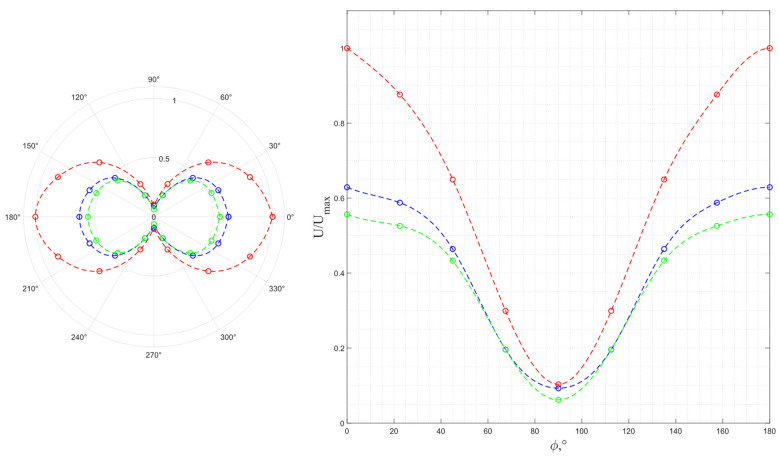
The experimental output voltage’s dependence on the relative orientation of the radiating and receiving piezo plates made of *X*-cut lithium niobate: red dot—4.7 MHz; blue dot—4.4 MHz; green dot—5.1 MHz.

The results of a study of the wave polarization for a pair of piezo plates made of *X*-cut lithium niobate are shown in Figure 21, where the dotted line is a function, |cos ϕ|. The *X* cut of LiNbO_3_ has the piezoelectric activity in two orthogonal directions so that the waves emitted by such a cut have an elliptical particle motion, which corresponds to the results of the previous numerical simulation. Therefore, the frequency dependance of the ratio of the ellipse axes is of interest. However, the experimental results show that the *X* cut of lithium niobate does not provide the generation of pure transverse waves, which corresponds to the absence of the output voltage zero value, at any frequencies.

Thus, the excitation of the linear polarized wave using this material and cut is impossible, which makes LiNbO_3_ *X*-cut piezoelectric plates unsuitable for use in the solid-state acoustic motion sensor under consideration despite their high efficiency, reaching a maximum at 4.7 MHz.

### 4.4. PZT-5H

**Figure 22:**
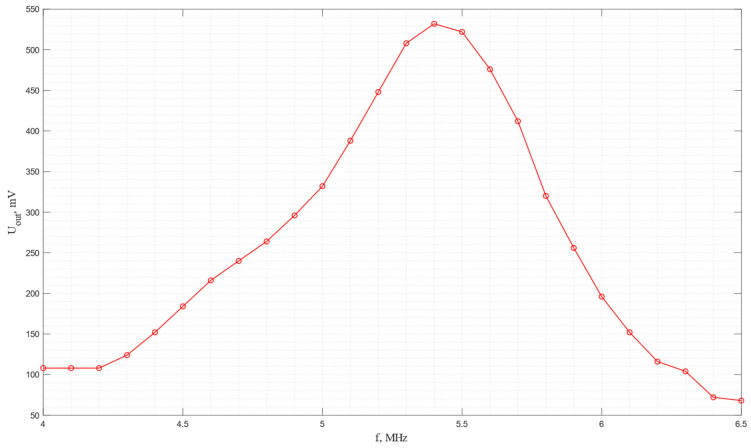
The frequency dependence of the output voltage for the acoustic system with receiving and emitting plates made of shear cut of PZT-5H.

The amplitude–frequency characteristic of the acoustic system in which a pair of piezo plates made of shear cut piezoceramics PZT-5H are used is shown in Figure 22. As seen from the graph, the maximum efficiency of electroacoustic transformation is observed at a frequency of 5.4 MHz.

**Figure 23:**
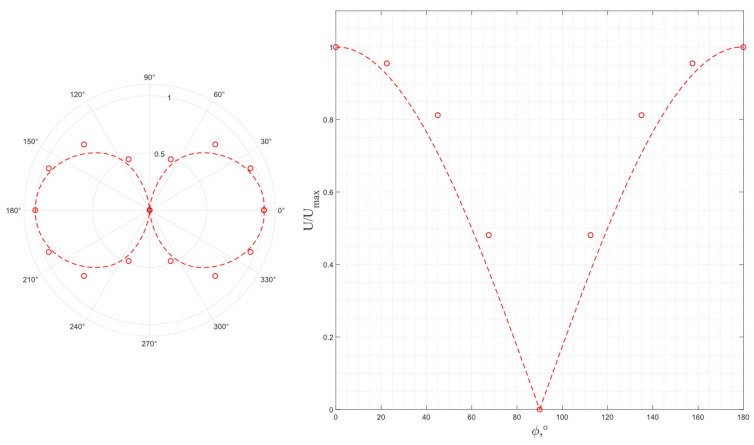
The experimental output voltage’s dependence on the relative orientation of the radiating and receiving piezo plates made of shear cut of PZT-5H.

The results of the study of the wave polarization for a pair of piezo plates made of a shear cut of piezoceramics of the type PZT-5H are shown in Figure 23, where the dotted line is a function, |cos ϕ|.

As seen from the graph, such plates make it possible to excite pure transverse waves with high efficiency, which is a crucial factor for the proposed bulk acoustic wave gyroscope design. However, the manufacture of high-frequency transducers from piezoceramics is a difficult process due to the high fragility of the material; moreover, at high frequencies, ceramics have low resistance, which leads to a decrease in the efficiency of electroacoustic conversion.

### 4.5. Frequency Characteristics of Piezodielectric Plates

**Figure 24:**
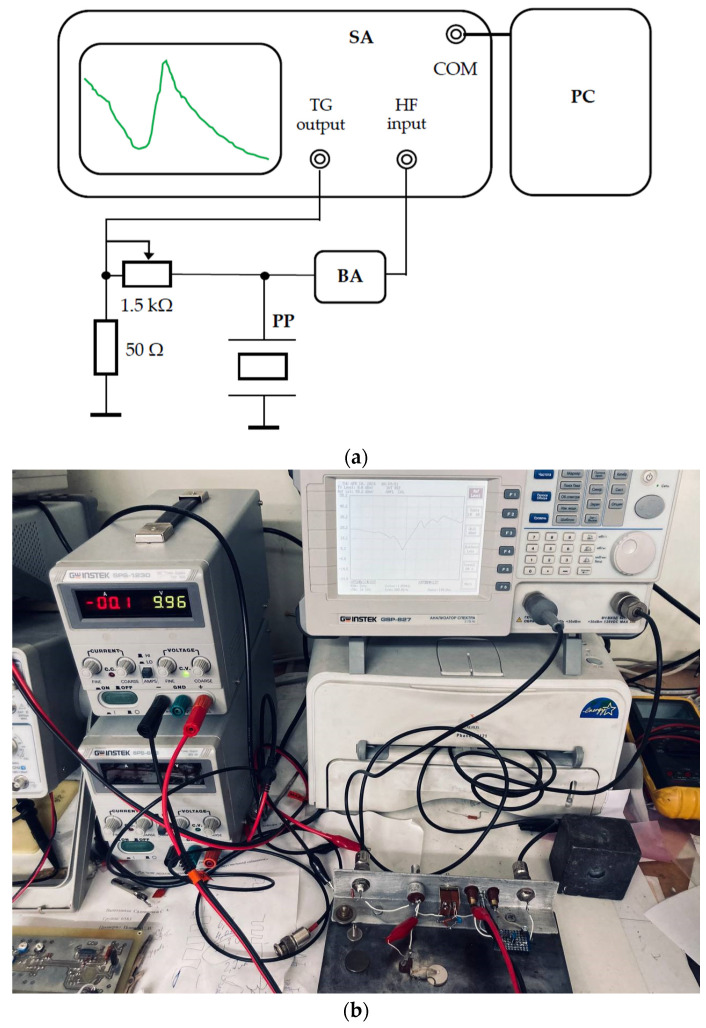
The experimental setup for measuring the piezo plate frequency characteristic: (**a**) block diagram of the experimental setup; (**b**) photograph of the experimental setup.

Next, the impedance frequency characteristics of the used plate piezoelectric transducers were measured to determine, experimentally, the velocities of transverse waves excited by these plates and to confirm the previously obtained calculated values. The block diagram of the experimental setup, used to research the impedance frequency characteristics, is shown in Figure 24.

A tracking frequency signal within predetermined limits is removed from the tracking generator (TG) output and fed through a variable resistor to the piezoelectric plate (PP) transducer under study. In this way, a voltage divider is formed and the voltage across the PP is proportional to its resistance modulus. The output voltage after the buffer amplifier (BA) goes to the high-frequency (HF) input.

This scheme allows not only to depict the impulse frequency characteristic (IFC) on the spectrum analyzer (SA) screen but also to transfer information to a PC for an accurate IFC comparison.

**Figure 25:**
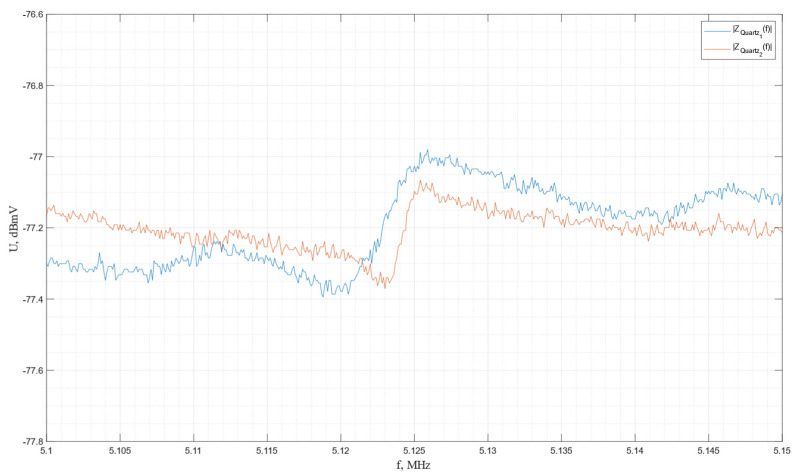
The impulse frequency characteristics of the piezo plates made of *Y*-cut piezo quartz.

The impulse frequency characteristics of the piezo plates made from *Y*-cut piezo quartz are shown in Figure 25.

In accordance with the graph, the resonance and antiresonance frequencies are determined to be *f*r = 5.121 MHz and *f*a = 5.126 MHz for the first plate and *f*r = 5.123 MHz and *f*a = 5.125 MHz for the second plate.

**Figure 26:**
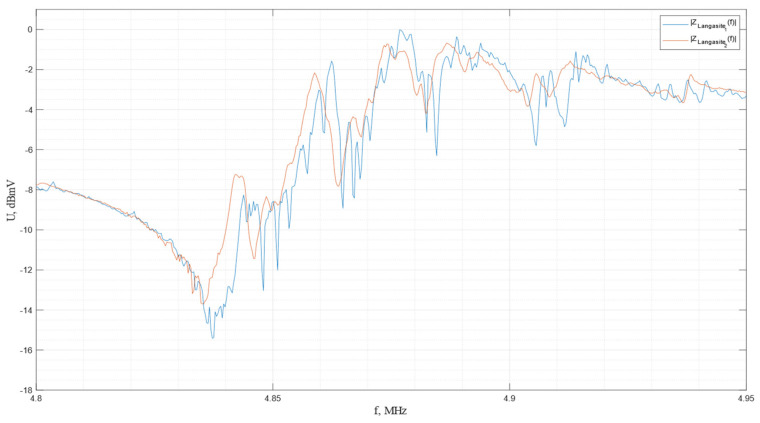
The impulse frequency characteristics of the piezo plates made of *Y*-cut langasite.

The impulse frequency characteristics of the piezo plates made from *Y*-cut langasite are shown in Figure 26.

In accordance with the graph, the resonance and antiresonance frequencies are determined to be *f*r = 4.837 MHz and *f*a = 4.877 MHz for the first plate and *f*r = 4.835 MHz and *f*a = 4.874 MHz for the second plate.

**Figure 27:**
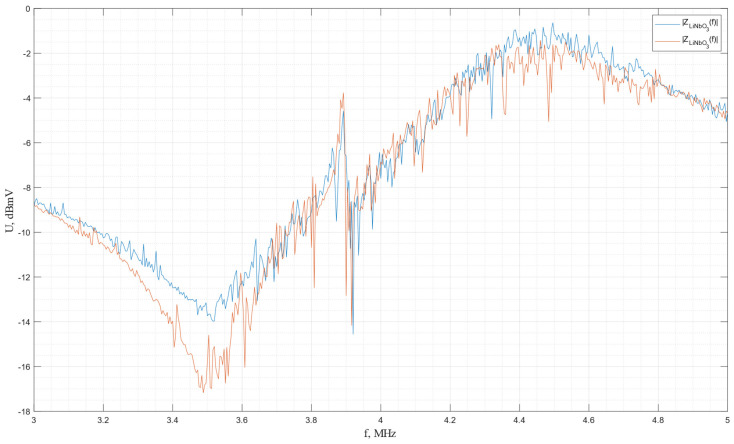
The impulse frequency characteristics of the piezo plates made of *X*-cut lithium niobate.

The impulse frequency characteristics of the piezo plates made from *X*-cut lithium niobate are shown in Figure 27.

In accordance with the graph, there are two frequencies of resonance and antiresonance for each piezo plate, which correspond to two different vibration modes for the *X*-cut lithium niobate, which confirms that the *X* cut of the lithium niobate plate excites two mutually orthogonal transverse waves. The resonance and antiresonance frequencies for the first plate are as follows: *f*r1 = 3.516 MHz, *f*a1 = 3.892 MHz, *f*r2 = 3.920 MHz, and *f*a2 = 4.496 MHz; for the second plate, they are *f*r1 = 3.488 MHz, *f*a1 = 3.892 MHz, *f*r2 = 3.916 MHz, and *f*a2 = 4.460 MHz.

**Figure 28:**
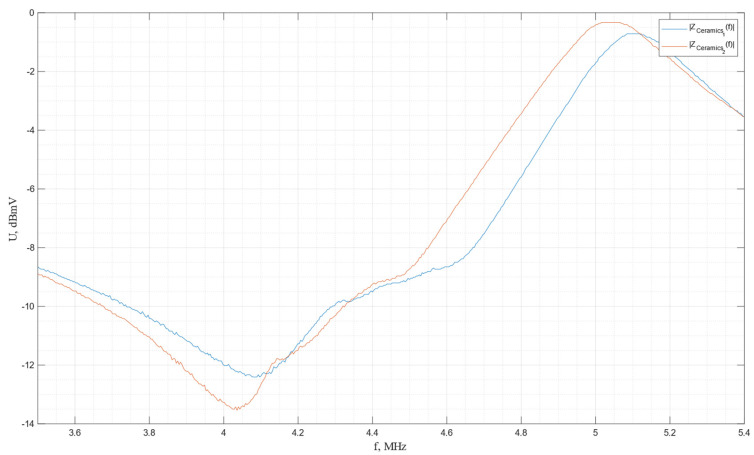
The impulse frequency characteristics of the piezo plates made of shear cut of PZT-5H.

The impulse frequency characteristics of the piezo plates made from a shear cut of PZT-5H piezoceramics are shown in Figure 28.

In accordance with the graph, the resonance and antiresonance frequencies are determined to be *f*r = 4.085 MHz and *f*a = 5.103 MHz for the first plate and *f*r = 4.036 MHz and *f*a = 5.039 MHz for the second plate.

The half-wave resonance of the piezo plate occurs under the following condition:

(3) d=λ/2,

Here, *d* is the plate thickness; λ is the wavelength in the medium.

The antiresonance frequency is related to the wavelength by the following expression:

(4) fa=v/λ,

Here, *f*a is the antiresonance frequency.

From Expressions (3) and (4), we obtain the dependence of acoustic wave velocity on the frequency and thickness of the plate:

(5) v=2dfa,

Taking into consideration the experimental values of antiresonance frequencies and measured plate thicknesses, using Formula (5), the velocities of transverse waves, *v*_exp_, excited by these plates can be determined and compared with the calculated ones, *v*_calc_, that is represented in Table 1.

Thus, the experimentally obtained velocities, *v*_exp_, of bulk acoustic waves converge with a high degree of accuracy with the values, *v*_calc_, calculated using the developed software, which confirms the validity of the numerical analysis method proposed by the authors.

## 5. Discussion

1. When exploring the sensing potential of a new solid-state acoustic motion sensor on bulk acoustic waves, one of the critical tasks is to increase its sensitivity, as shown by the previous research [14,15,16]. Based on the results of theoretical analysis, the principle of increasing the sensing potential by improving the electroacoustic conversion efficiency of the piezoelectric plate transducers by selecting optimal materials and cuts was formulated in this research.
2. The present paper provides the results of comprehensive theoretical, numerical, and experimental research studies conducted according to the formulated principles.
3. The method of efficiency analysis was proposed by estimating the following parameters: the value of the acoustic wave propagation velocity, the coefficient of the electromechanical transformation, and the angle between the quasi-longitudinal wave polarization vector and its propagation direction. The last one is critically important to estimate to ensure the excitation of only one pure transverse wave, which is the required condition of new solid-state acoustic motion sensor operation.
4. Numerical analysis was performed for the abovementioned parameters, and corresponding graphical dependencies were built for a number of materials and cuts: the *Y* cut of piezo quartz, *Y* cut of langasite, *X* cut of lithium niobate, and shear cut of PZT-5H. Taking into account the problem of calculation with relation to singularity points with a pair of eigenvalues equal to each other, a novel algorithm was developed that overcame an error in matching the phase velocity and polarization of the excited waves and subsequently ensured the operation of the piezoelectric plates and the sensor in general.
5. Two series of experimental studies were carried out to confirm the numerical analysis results: the first one showed a high correlation with the numerical estimation of wave polarization and efficiency; the second one showed a high correlation with the numerical estimation of wave propagation velocities, thus validating the proposed method.
6. Based on the results of theoretical analysis, numerical modeling, and experimental studies, *Y*-cut piezo quartz, *Y*-cut langasite, and shear cut ceramics were considered promising to be used as emitting and receiving piezo plates of novel bulk acoustic wave gyroscopes. However, piezo quartz has relatively low efficiency, piezoceramics are difficult to process in order to achieve high operating frequencies, and *X*-cut lithium niobate piezo plates are highly efficient but are not suitable due to the elliptical polarization of the emitted waves at any frequencies.
7. Thus, *Y*-cut langasite is proposed as an optimal material to increase the sensing potential of the considered new solid-state acoustic motion sensors.
8. The challenge for future scientific inquiry is to search for novel principles of constructive implementation and informative signal selection in order to increase sensing potential. One of the most promising directions is the solution of the problem of direct piezo plate radiation under rotation for the possible design simplification of the next generation of acoustic solid-state motion sensors on bulk acoustic waves.

## Figures and Tables

**Table 1:** The experimental results.

Material	*d,* mm	*f*a, MHz	*v*_exp_, mm/μs	*v*_calc_, mm/μs	Δ*v*, %
Piezo quartz	0.38	5.126	3.896	3.915	0.5
	0.38	5.125	3.895	3.915	0.5
La_3_Ga_5_SiO_14_	0.28	4.877	2.731	2.741	0.4
	0.28	4.874	2.729	2.741	0.4
LiNbO_3_	0.52	3.892	4.048	4.058	0.2
		4.496	4.676	4.806	3.0
	0.52	3.916	4.072	4.058	0.3
		4.460	4.638	4.806	3.0
PZT-5H	0.22	5.103	2.245	2.372	5.0
	0.22	5.039	2.217	2.372	6.0

## Data Availability

The data presented in this study are available on request from the corresponding author.
